# Porous ionic liquids: synthesis and application

**DOI:** 10.1039/c5sc01374g

**Published:** 2015-05-22

**Authors:** Shiguo Zhang, Kaoru Dokko, Masayoshi Watanabe

**Affiliations:** a Department of Chemistry and Biotechnology , Yokohama National University , 79-5 Tokiwadai , Hodogaya-ku , Yokohama 240-8501 , Japan . Email: mwatanab@ynu.ac.jp

## Abstract

Solidification of fluidic ionic liquids into porous materials yields porous ionic networks that combine the unique characteristics of ionic liquids with the common features of polymers and porous materials. This minireview reports the most recent advances in the design of porous ionic liquids. A summary of the synthesis of ordered and disordered porous ionic liquid-based nanoparticles or membranes with or without templates is provided, together with the new concept of room temperature porous ionic liquids. As a versatile platform for functional materials, porous ionic liquids have shown widespread applications in catalysis, adsorption, sensing, actuation, *etc.* This new research direction towards ionic liquids chemistry is still in its early stages but has great potential.

## Abstract 2

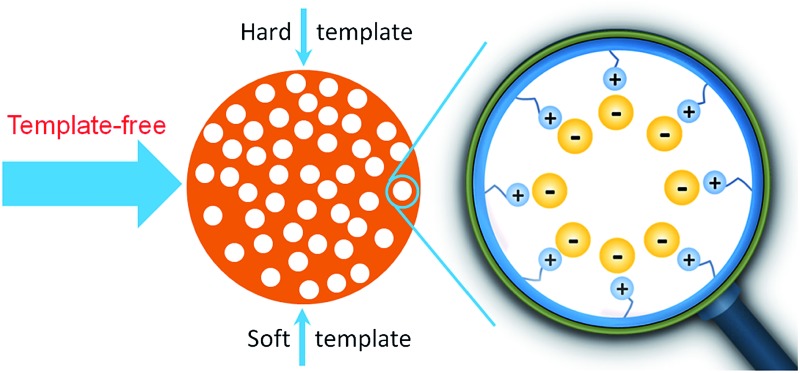
Porous ionic liquids combine the unique characteristics of ionic liquids with the common features of polymers and porous materials.

## Introduction

Since the discovery of air- and water-stable ionic liquids (ILs) by Wilkes in 1992,^1^ the research and development (R&D) of ILs has attracted the overwhelming interest of both scientists and engineers.^2^–^9^ ILs have unique and tunable properties compared with traditional molecular compounds including a wide liquid range, high ionic conductivity, negligible volatility, non-flammability, high electrochemical and thermal stability, as well as good solvation ability. Such properties have led to an explosion in their use as green, designable solvents for a variety of processes linked to green chemistry and clean technology (*e.g.*, catalysis and organic synthesis),^2^,^7^,^10^ as ideal electrolytes for electrochemistry and energy-related applications (*e.g.*, lithium batteries, fuel cells, supercapacitors, and dye-sensitized solar cells).^3^,^11^ And as versatile materials for advanced applications in material sciences and engineering.^5^,^12^ However, the fluidity, high viscosity, and slow gas diffusivity of the liquid-like ILs strongly limit their applications, recyclability, and separation.

**Fig. 1:**
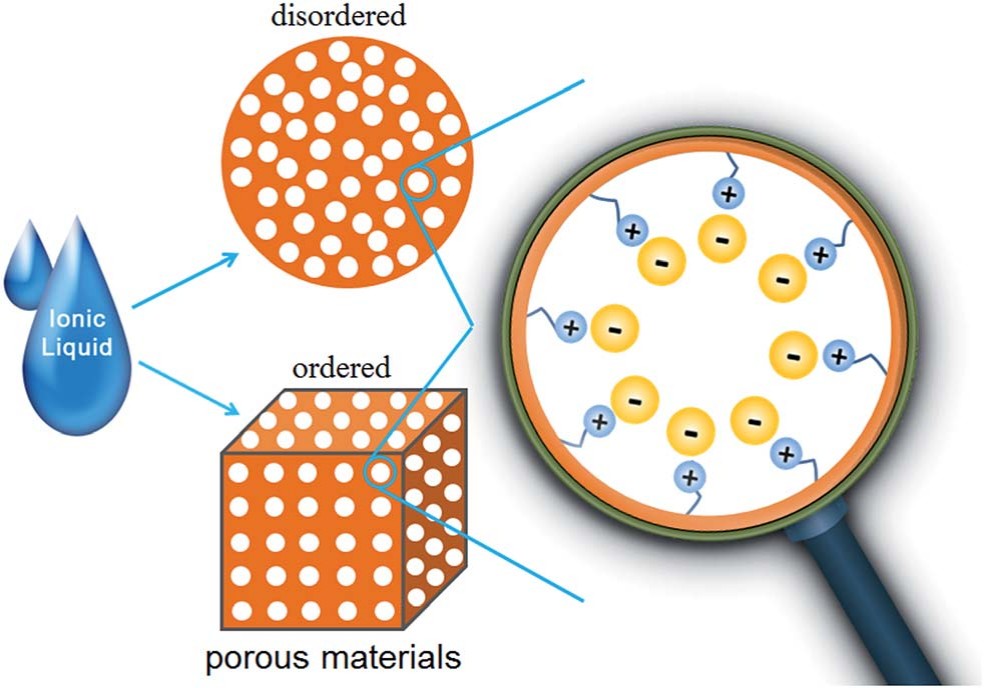
Solidification of ILs into ordered or disordered porous materials.

Solidification of fluidic ILs into porous materials yields porous ILs that integrate the unique characteristics of ILs into common polymers and porous materials (Fig. 1). Porous ILs possess low bulk density, enlarged surface areas and pore volumes, and combine the chemical versatility of ILs with the spatial architecture, enhanced mechanical stability, and processability inherent to polymeric architectures.^13^–^15^ Owing to the intrinsically charged character within their pores, porous ILs can alter their physicochemical properties much more broadly and easily than common non-ionic porous polymers by counterion exchange, an effect well known to the IL community. The high ionic density, together with the high polarizability and strong electrostatic field, provide a special local chemical environment inside the pores that has never been achieved before in molecular porous materials, is responsible for the unusual properties and performance of ILs, and is expected to amplify their function. In addition, compared to the so-called supported ILs, wherein ILs are covalently grafted on the pore surface of rigid inorganic hosts such as silica,^16^,^17^ the all-organic porous ILs are of special interest because of the variability of their monomeric building blocks, as well as their flexible ionic skeletons. This “soft” feature for porous ILs makes it possible to fabricate advanced bulk materials such as smart membranes. In the following, we present recent advances in the field of porous ILs, with a focus on template and template-free synthesis, as well as their potential applications.

## Templating synthesis of porous ILs

### Hard templates

As with porous inorganic materials, for which sophisticated templating methods are intensively employed to introduce pores, porous ILs are generally obtained by *in situ* polymerization of IL monomers or copolymerization of ILs with other monomers in the presence of hard templates.^18^–^22^ For example, Wilke *et al.* successfully fabricated mesoporous ILs by backfilling the interstitial voids of porous colloidal crystal silica with an IL monomer solution followed by cross-linking and etching. The resulting porous ILs featured an enlarged surface area (150–220 m^2^ g^–1^), a well-defined and uniform pore shape, a narrow pore size distribution, and enhanced CO_2_ adsorption. It is noteworthy that a high degree of cross-linking was necessary to stabilize the polymer mesoporous structure against collapse after the removal of the silica template.^18^

**Fig. 2:**
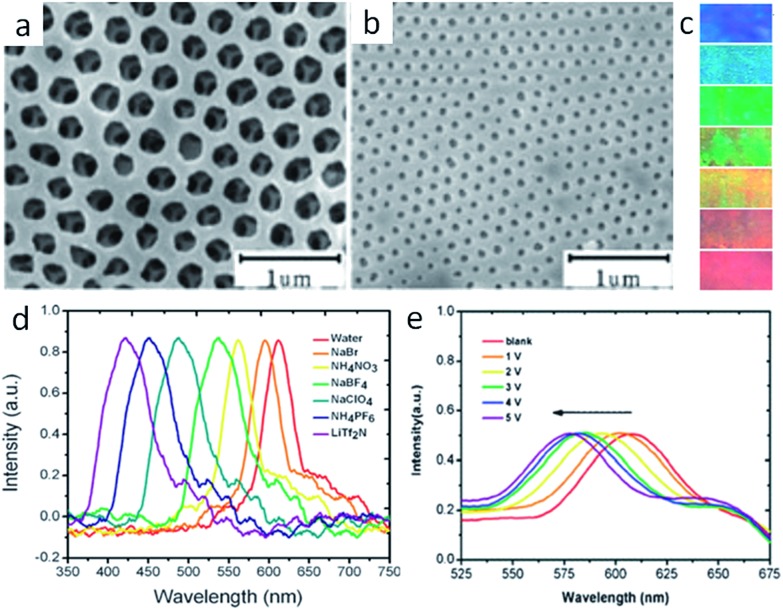
SEM images of the photonic IL films with (a) [Br] or (b) [Tf_2_N] anions. Anion-exchange-induced (c) color change and (d) stopband shifts of the photonic films. (e) Stopband shifts of the photonic film with an increase in applied voltage. Adapted with permission from ref. 21 Copyright 2010, the Royal Society of Chemistry.

When large, ordered silica colloidal crystal arrays are used as the hard template, three-dimensional (3D) ordered and interconnected macroporous ILs (photonic ILs) are achieved in an inverse opal structure of a face-centered-cubic template (Fig. 2).^19^–^22^ A three-step approach was involved for the preparation of such porous ILs: fabrication of the silica colloidal crystal template; infiltration of the template with a mixed solution of IL monomer, cross-linker, and initiator, followed by polymerization; and selective dissolution of the SiO_2_ template. The highly ordered and periodic macroporous nanostructures directly generate optical signals due to Bragg diffraction, as observed for photonic crystals.^23^ For the normal incidence geometry, the Bragg reflection wavelength (stopband, *λ*) of the inverse opal is determined by the lattice constant and the refractive index of the polymer network in the 3D ordered inverse opal structure using the following equation, *λ* = 2*d*(0.74 + 0.26*n*^2^)^1/2^, where *d* is the characteristic spacing, and *n* is the refractive index of the polymer network.^24^ As diversification of the counteranions causes different degrees of shrinking of the prepared pontonic IL film, and thus a decrease in parameter *d*, the ionic nature of the pontonic ILs offers an additional possibility to tune the stopband of photonic crystals *via* an anion exchange-induced volume change. As shown in Fig. 2a and b, it is clear that the macropores, as well as the characteristic small window channels connecting the neighboring macropores in the [Br]-based film, become closed after ion exchange with [Tf_2_N]. Correspondingly, the stopband shifts gradually to a shorter wavelength range with the direct exchange of [Br] by more hydrophobic anions such as [NO_3_], [BF_4_], [ClO_4_], [PF_6_], and [Tf_2_N] (Fig. 2d). In concert with the *ca.* 200 nm shift of the stopband, the photonic IL film substantially changes from red to blue (Fig. 2c). As a result, the anion exchange events can be transformed into readable optical signals (color change).^21^ In addition to optical anion recognition and sensing, the photonic ILs can also function as a new type of optical sensory material in “naked-eye” detection of humidity, as well as for solvents with different polarities.^19^,^20^,^22^ More interestingly, the stopband of the photonic ILs is very sensitive to an external electric field. With an increase in the applied voltage, the reflection peak of the photonic IL film containing [BF_4_] anions clearly shifts toward a shorter wavelength, with a shift of about 30 nm for a field of *ca.* 5 V (Fig. 2e). This response is very fast, on the order of seconds, and is reversible.^21^ Although this phenomenon is peculiar to ILs and has not been addressed, this result indeed demonstrates the great potential of photonic ILs in electro-optic switches.

**Fig. 3:**
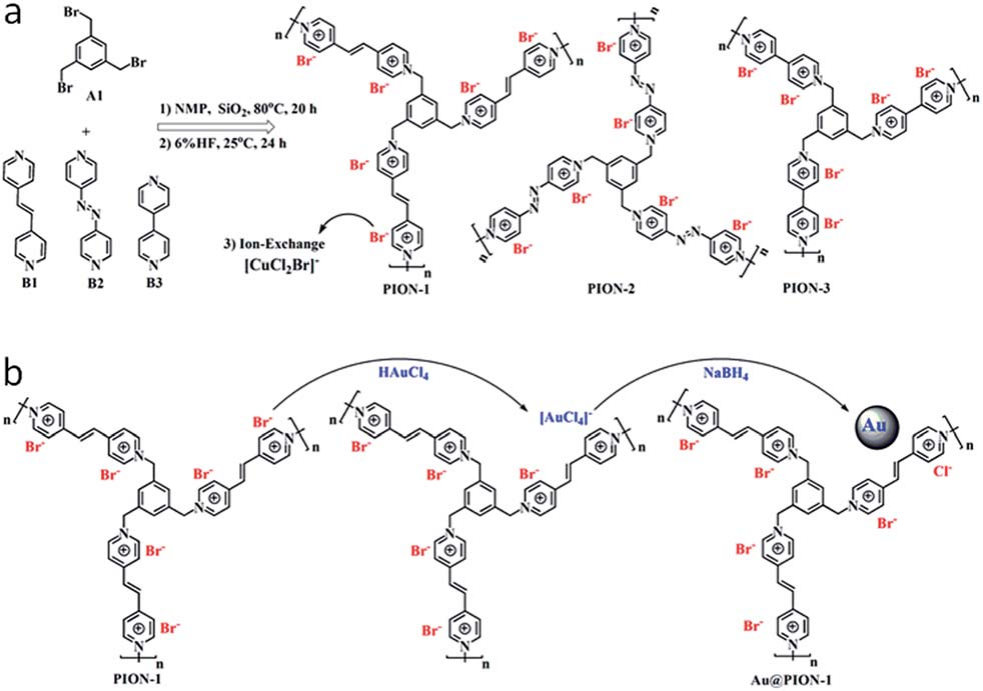
(a) Synthetic routes to nanoporous IL networks through condensation polymerization. (b) A controllable route to Au-supported hybrid materials through simple anion exchange and chemical reduction. Reproduced with permission from ref. 25 Copyright 2015, American Chemical Society.

Instead of conventional radical polymerization of monomeric ILs, nanoporous IL networks can also be achieved through condensation polymerization between commercial 1,3,5-tris(bromomethyl)-benzene (A1) and 1,2-di(4-pyridyl)ethylene (B1), 4,4′-azopyridine (B2) or 4,4′-bipyridine (B3) *via* S_N_2 nucleophilic substitution in the presence of a hard template (12 nm silica particles) (Fig. 3a).^25^ Nitrogen sorption isotherms showed that all of the obtained porous ILs had specific surface areas of 107–132 m^2^ g^–1^, as well as a hierarchical pore structure dominated by large mesopores and macropores. Importantly, porous ILs with a highly ionized backbone, owing to the electrostatic and steric effect, can stabilize and support quite small (1–2 nm) Au nanoparticles through simple anion exchange and chemical reduction (Fig. 3b). The resulting Au/PION hybrid materials serving as a heterogeneous catalyst are highly active, selective, and stable in the aerobic oxidation of saturated alcohols. For example, an exceptional activity with remarkable turnover frequency (TOF) of 2064 h^–1^ was achieved for the selective oxidation of cyclohexanol to cyclohexanone.

### Soft templates

In contrast to hard templates, soft templates, which have been widely used in the fabrication of inorganic porous materials, are rarely applied in research on porous ILs because of the difficulty in template removal that relies on solvent extraction.^26^ Balancing the interaction between template and organic precursor is crucial to enable polymerization around the template with suitable binding force, while at the same time avoiding close conjunction between the soft template and organic precursors, thus allowing removal of the template by solvent extraction. Tri-block copolymer, EO_20_PO_70_EO_20_ (P123), has been successfully utilized as a single soft template to fabricate hierarchical meso-macroporous monolithic ILs by radical self-polymerization of the 1-allyl-3-vinylimidazolium monomer.^27^ The key point is that P123 and IL monomers were initially dissolved in water with vigorous stirring to form well-dispersed micelles, which were expected to achieve sufficient interaction and thermal equilibrium between the IL cationic precursor and the soft template through the S^0^H^+^X^–^I^+^ mode (S^0^: nonionic surfactant P123, H^+^: hydrogen ions of ionization, X^–^: IL anion, I^+^: IL cation) based on the charge matching principle.^28^ Porous ILs resulted after removing the soft template by extraction in ethanol. Depending on the synthetic parameters, including the template concentration and the anions of the IL precursor, the surface area of the obtained porous ILs could be tuned to as high as 144 m^2^ g^–1^. The porous architecture was stable to undergo anion exchange for further functionalization. For example, when the guest anions on the pore walls were replaced by a catalytically active anion, polyoxometalate, the resulting porous ILs could serve as recyclable heterogeneous catalysts that showed superior activity in the liquid-phase epoxidation of *cis*-cyclooctene with H_2_O_2_.^27^

## Template-free synthesis of porous ILs

### Radical copolymerization of IL monomers and cross-linkers

**Fig. 4:**
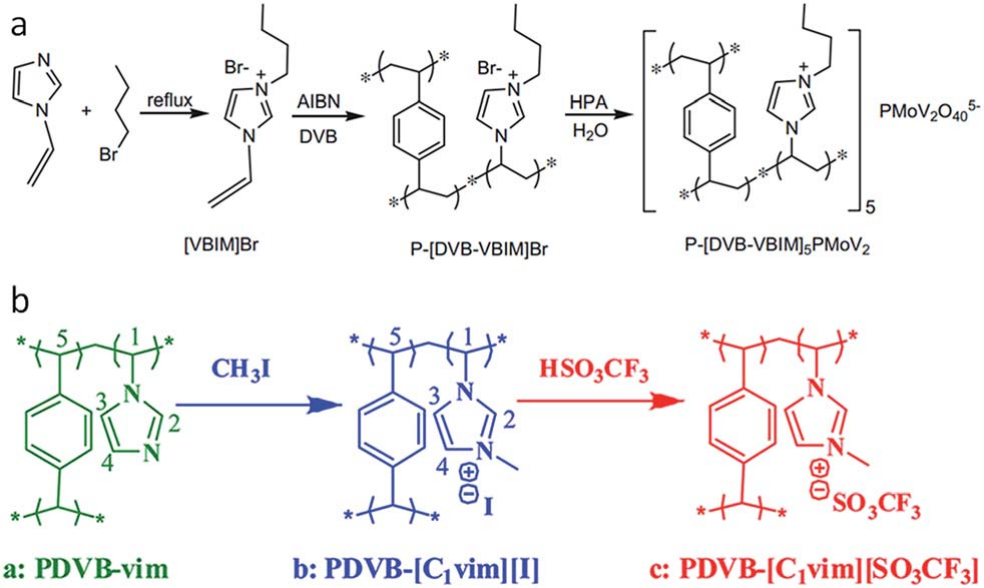
Template-free synthesis of porous ILs: (a) radical copolymerization of the IL monomers and cross-linker divinylbenzene. Reproduced with permission from ref. 29 Copyright (2012), Elsevier. (b) Quaternary ammonization of the as-synthesized porous polymers with following anion-exchange. Reproduced with permission from ref. 30 Copyright 2012, American Chemical Society.

Despite the great potential, the template-based strategies require sacrificial components, and are time and energy consuming, suffering from tedious synthetic procedures and small surface areas (<220 m^2^ g^–1^). Furthermore, both hard and soft templates must be removed by an additional etching or solvent extraction step to yield a porous structure, while calcination usually causes damage to the ionic framework before the template is removed. In contrast, a template-free approach would greatly shorten the synthetic period and reduce the overall cost. It was recently found that direct radical copolymerization of the IL monomers (3-alkyl-1-vinylimidazolium bromide) with cross-linker, divinylbenzene (DVB), gives rise to intrinsically mesoporous ILs without adding stabilizers, surfactants, hard or soft templates (Fig. 4a).^29^,^31^,^32^ Depending on the molar ratio of ILs to DVB, the solvent type and volume, and the alkyl chain length of ILs, the surface area of the porous ILs are easily tuned in the range of 52–649 m^2^ g^–1^.^32^ The obtained porous ILs have shown potential applications in CO_2_ adsorption^32^ and catalysis after incorporation of functional anions (*e.g.*, heteropolyanion in Fig. 4a)^29^ or cations.^31^ For example, functionalization of the IL monomer by an amino–alkyl chain yields a porous IL with a high surface area of 429 m^2^ g^–1^ after ion exchange with NaOH.^31^ Because of the unique dual Lewis–Brønsted basic sites (R-NH_2_ and OH^–^), as well as the highly porous structure, this porous IL exhibits good catalytic performance in solvent-free Knoevenagel condensation with high activity and selectivity, steady reusability, applicability to various substrates, and resistance to CO_2_/water contamination.

Distinct from the one-step copolymerization method, porous ILs can also be obtained by copolymerization of 1-vinylimidazolate (vim) with DVB, followed by quaternary ammonization and anion-exchange treatment, as shown in Fig. 4b. Not only the surface area, but also the wettability of the reactants can be tuned by the quaternary ammonization and anion-exchange. For example, the surface area of PDVB–vim (670 m^2^ g^–1^) was greatly reduced to 592 m^2^ g^–1^ and 181 m^2^ g^–1^ for PDVB–[C_1_vim][I] and PDVB–[C_1_vim][SO_3_CF_3_], respectively. PDVB–[C_1_vim][SO_3_CF_3_], as an efficient heterogeneous catalyst, exhibited even higher activities than the corresponding homogeneous ILs in a series of catalytic reactions, such as transesterification, Peckmann reaction, Kharasch addition, esterification, and hydration. This is probably because the reactants (*e.g.*, methanol and tripalmitin) have lower contact angles and thus much better wettability on the mesoporous IL than the products (*e.g.*, methyl palmitate and glycerol).^30^

### Trimmerization of nitrile-containing ILs

**Fig. 5:**
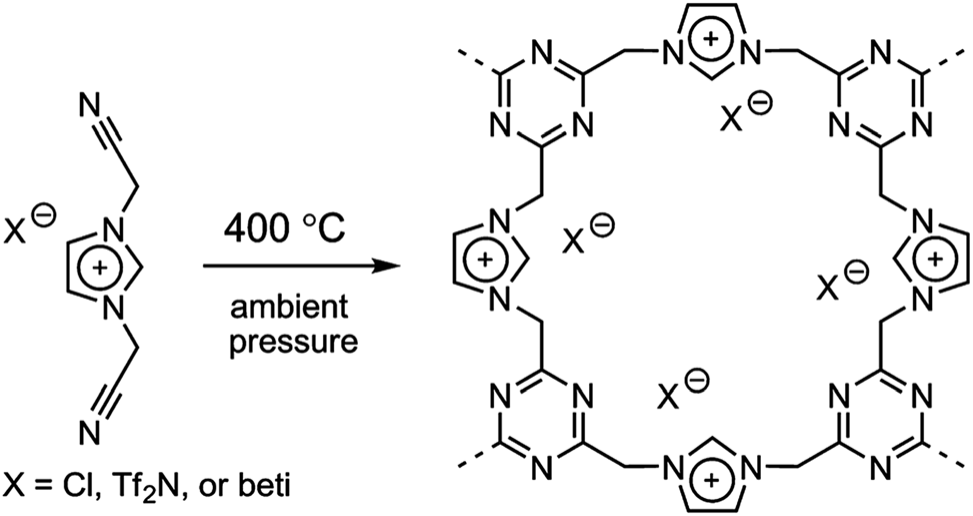
Template-free synthesis of porous ILs by direct trimerization of nitrile-containing ILs. Reproduced with permission from ref. 33 Copyright 2009, American Chemical Society.

It was recently demonstrated that direct carbonization of ILs with nitrile-bearing cations yielded porous carbons.^34^ However, at lower temperature such as 400 °C, an amorphous porous polytriazine network apparently forms through cyclotrimerization reactions between nitrile groups on neighboring cations, while a significant fraction of anions presumably remains,^33^,^35^ as shown in Fig. 5. The anion structures were found to strongly affect the resulting surface areas. Generally, porous polymers derived from bulky anions feature large surface areas and pore volumes. This is mainly because of the templating role of the anions, which may partially decompose during trimerization of nitrile groups. When the anion was changed from [Cl] to [Tf_2_N] and [beti], the resultant surface area varied from 2.0 m^2^ g^–1^ to 481 m^2^ g^–1^ and 814 m^2^ g^–1^. In addition to the large and tunable surface area offered by these porous ILs, the constituent anions were ion-exchangeable. The potential applications of these anion-exchangeable porous ILs were disclosed by the adsorption of perrhenate ions (ReO_4_^–^) in aqueous solution. The uptake of ReO_4_^–^ is primarily driven by ion exchange and not by physical adsorption, as the observed trend for adsorption capacity is completely different from that of the surface area. For example, the [Cl]-derived porous IL exhibited much higher capacities than those of the bulkier anions because the residual amount of anion in this case is far greater than for [Tf_2_N] and [beti].

### Facile ionic complexation between poly(ionic liquids) and acids

**Fig. 6:**
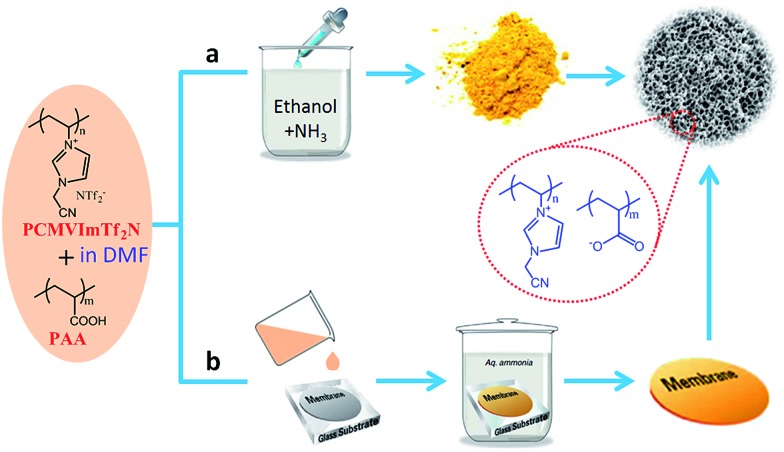
Template-free synthesis of porous ILs in the state of (a) nanoparticles or (b) membranes *via* ionic complexation.

All of the methods mentioned above require long reaction times and controlled polymerization steps in the presence or absence of templates. Consequently, straightforward synthetic strategies that provide porous ILs are highly desirable. Very recently, a template-free and easily scalable strategy to prepare porous ILs was developed by Yuan *et al.* through simple ionic complexation between the as-synthesized poly(ionic liquid)s (PILs) and polyacids triggered by deprotonation of the carboxylic acid groups.^36^–^45^ As shown in Fig. 6a, poly(3-cyanomethyl-1-vinylimidazolium bis(trifluoromethylsulfonyl)imide) (PCMVImTf_2_N) and poly(acrylic acid) (PAA) are first dissolved in a good solvent such as DMF to form a homogeneous, transparent solution. The molecular-level mixing solution is then added drop wise into an excess of alkaline solution such as ethanol containing 0.5 wt% of NH_3_. Turbidity and insoluble aggregates form immediately *via in situ* deprotonation of the carboxylic acid (COOH) groups of PAA chains to form carboxylate groups (COO^–^) and ionic complexation between PCMVImTf_2_N and deprotonated PAA. Micro-mesoporous IL nanoparticles with high surface areas (330 m^2^ g^–1^) and large pore volumes (1.10 cm^3^ g^–1^) are obtained after removing the placeholder solvents. It is expected that the strong ionic complexation and the high ionic cross-linking density of the system solidify and stabilize the polyelectrolyte network with minimized chain mobility. The resulting porous ILs remain stable in some organic solvents, even under reflux treatment. This template-free method can be readily extended to other PILs with variable cations or anions.^36^ Several processing parameters, including the ratio of two charged species, the polymer concentration, and the types of complexation solvents, seem to affect the degree of ionic complexation and thus play a dominant role in controlling the porous structure.^36^–^42^ Further changing the PAA into carboxylic acid-substituted pillar[5]arene (C-pillar[5]arene)^40^ or small-molecule multivalent carboxylic acids,^38^ or combining the imidazolium cations and the carboxylic acid units into the same polymer chain^39^ or even the same IL moiety (carboxylic acid functionalized zwitterionic PILs)^43^ can also crosslink PILs into porous polyelectrolyte frameworks. The synergistic effect between ILs, polymers, and porous materials endows the micro-mesoporous ILs with great potential for controlled capture or separation of CO_2_,^39^ catalysis,^36^,^43^ efficient removal of organic dye molecules,^38^ selective adsorption of alkylene diols,^40^*etc.* In particular, by combining the high surface area and strong electrostatic environment, the porous ILs having a uniform distribution of functional groups could serve as robust catalyst supports with abundant anchoring points to immobilize copper salts in an unconventional ion pair binding mode. The resulting hybrids are highly efficient as heterogeneous catalysts for the aerobic oxidation of hydrocarbons under mild conditions.^36^

Following the above dissolution–precipitation strategy, but slightly modifying the fabrication method, Yuan *et al.* further succeeded in constructing porous IL membranes using a simple film casting and solution immersion/activation procedure.^37^,^41^,^42^ As depicted in Fig. 6b, the homogeneous solution of hydrophobic PIL and polyacid is first cast onto a glass plate and dried to form a membrane. Then the membrane is soaked in an alkaline aqueous NH_3_ solution in order to charge the acid moieties and activate interchain ionic bonding and structural rearrangements between the polyacid and surrounding cationic PILs, thus developing pores within the membrane on the nanoscale level. A freestanding but flexible membrane that is easily detached from the glass substrate is obtained. After coating onto an optical fiber *in situ*, the porous ionic membranes, being simultaneously charged and nanoporous in nature, showed superior pH sensing performance with a fast response and high sensitivity.^37^ In addition, the mechanical strength of the porous IL membranes was reinforced by grafting them onto cellulose nanofibrils. The Young's modulus and stress at failure after reinforcement were found to increase from ∼470 to ∼610 MPa and from ∼7.8 to ∼10.4 MPa, respectively.^44^

**Fig. 7:**
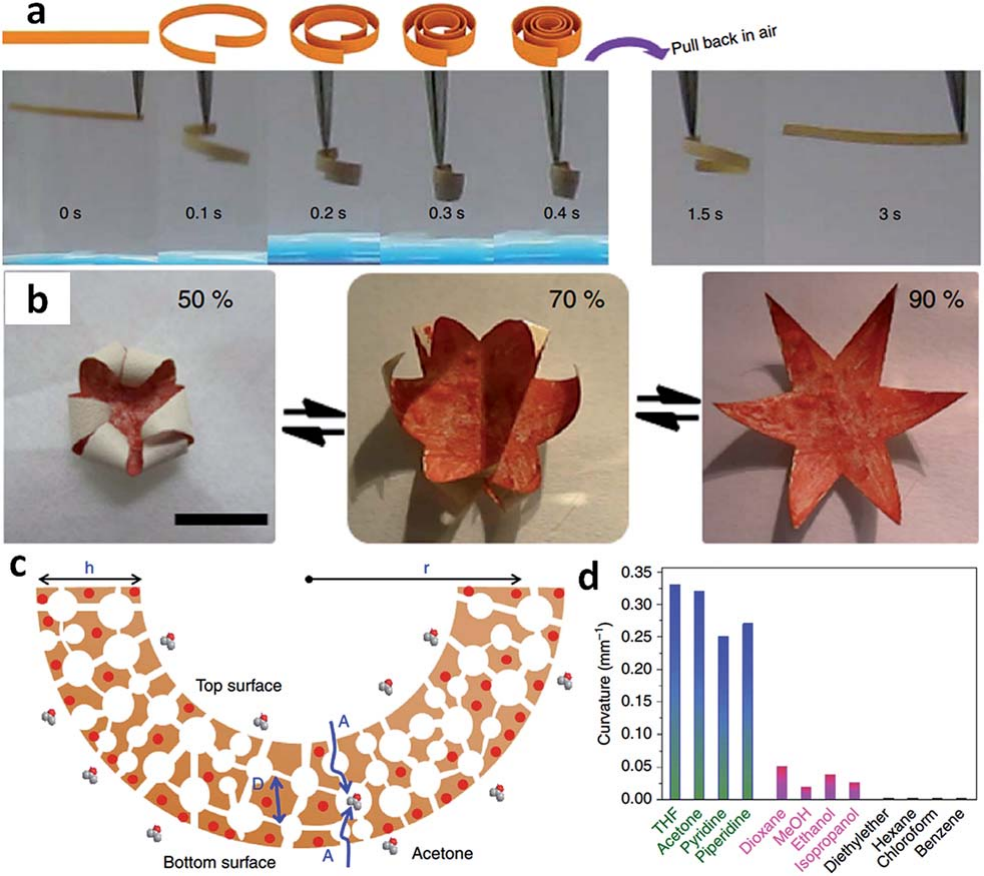
(a) Adaptive movement of a PILTf_2_N/C-pillar[5]arene membrane placed in acetone vapor (24 kPa, 20 °C, left) and then back in air (right). (b) Reversible closing and opening of a star-shaped membrane actuator ‘flower’ upon switching the humidity between 50% and 90% at 20 °C. (c) Diffusion of acetone–vapor molecules throughout the porous membrane and preferentially interaction between acetone and [Tf_2_N] anions. (d) Curvatures of PILTf_2_N/C-pillar[5]arene membrane actuator in different solvent vapors. Adapted with permission from ref. 41 Copyright 2014, Macmillan Publishers Ltd.

The structure of acids was found to greatly affect the distribution of pore sizes in the bulk membrane. For example, porous membranes obtained from PAA exhibited a hierarchical, two-zone pore structure consisting of a thin layer of surface macropores in addition to a thick layer of 3D interconnected nanopores (30–100 nm). Replacing PAA with high-acidity multivalent benzoic acid derivatives resulted in porous membranes bearing an interesting gradient in both cross-linking density and pore size along the cross-section.^42^ A similar gradient structure was also observed when using C-pillar[5]arene as the acid source. A gradient in the degree of electrostatic complexation (DEC) formed along the cross-section of the membrane, as demonstrated by quantitative determination of the released [Tf_2_N] anions in solution during complexation.^41^ The [Tf_2_N] content was inversely proportional to the DEC and increased in the top-down direction, that is, the top surface had a lower [Tf_2_N] content and thus a higher DEC than the bottom. This gradient architecture arises from the soaking step, during which ammonia diffuses into the membrane from top to bottom, deprotonates the COOH and simultaneously triggers the electrostatic complexation. Very interestingly, the PILTf_2_N/C-pillar[5]arene membranes consisting of interconnected pores (200 nm to 3 μm) exhibited a reversible and repeatable response towards a variety of organic vapors with very fast actuation speed, large-shape deformation, and robust responsiveness. As shown in Fig. 7a, when exposed to acetone vapor, the flat membrane bends quickly into a closed loop in ∼0.1 s, and further into a tightly wound coil in 0.4 s. The membrane can recover its original flat shape rapidly in ∼3 s upon re-exposure to air. Furthermore, the porous IL membrane also readily exhibits hygroscopic actuation. A star-shape hygroscopic actuator can even mimic an “artificial flower“ that blossoms and closes in response to humidity changes, indicating the ability of cooperative actuation to “enwrap“ objects (Fig. 7b).

Compared to traditional hydrogel and ion gel actuators whose modulation is limited by slow diffusion in the wet state, the porous IL membrane that bend in response to acetone vapor shows unprecedented responsiveness, with a large-scale locomotion and speed of an order of magnitude faster than the state-of-the-art. This is ascribed to the unique combination of porous morphology and gradient structure of the membrane, as well as the interaction between solvent molecules and ILs. As illustrated in Fig. 7c, when the membrane is exposed to acetone vapor, the solvent molecules diffuse rather rapidly into the porous membrane from both directions (top and bottom) and preferentially interact with the [Tf_2_N] anions. Because the membrane possesses asymmetrical DEC distribution, and thus a gradient concentration of [Tf_2_N] anions in its cross-section, the bottom surface is more solvated by acetone molecules compared with the top, which gives rise to a greater swelling and volume change in the bottom portion and leads to actuation movement. In addition, the porous architecture with submicron-scale interconnected pore channels greatly accelerates the actuation speed by providing instant access to the pore walls throughout the membrane. The solvent type, and thus the “solvent-PILTf_2_N” interaction, strongly affects the amplitude of actuation. For example, unlike acetone, dioxane, which cannot dissolve but can swell the PILTf_2_N polymer, drives the actuator with slower kinetics and weaker bending, while diethyl ether, which can neither dissolve nor swell the PILTf_2_N polymer, fails to drive the actuator (Fig. 7d).

**Fig. 8:**
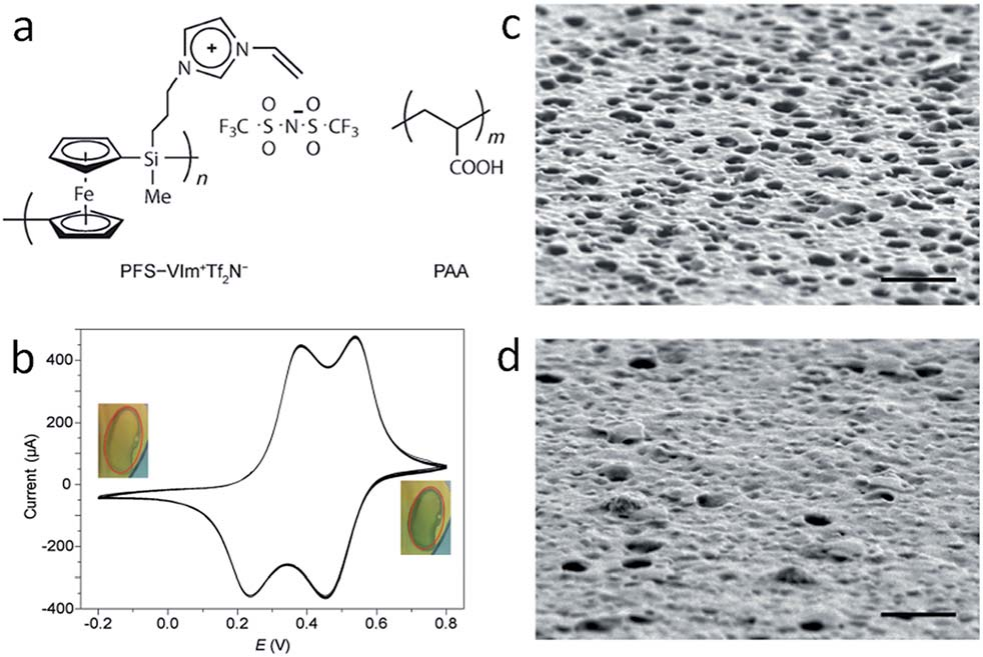
(a) Redox-responsive porous membranes formed by complexation between redox active PFS–VImTf_2_N and PAA. (b) Cyclic voltammograms of PFS–VImTf_2_N/PAA complex membrane on Au substrate at a scan rate of 5 mV s^–1^. Surface SEM images of porous membranes after oxidation at 0.6 V for 10 min (c), and then reduction at –0.2 V for 10 min (d). Scale bar: 1 mm. Reproduced with permission from ref. 46 Copyright 2014, Wiley.

Besides the intrinsic actuator ability, advanced functional porous IL membranes can provide a unique response after incorporating additional stimuli-responsive groups into the polymeric architecture. For example, redox-responsive porous membranes can be readily formed by electrostatic complexation between redox active poly(ferrocenylsilane) (PFS)-based PIL (PFS-VImTf_2_N) and PAA (Fig. 8a).^46^ After coating this porous membrane onto the surface of an electrode, electrochemical oxidation of the redox active ferrocene units along the PFS chains in the porous membrane is realized, as evidenced by the double-wave voltammogram, as well as a color change from amber to green-blue, which returns to amber upon reduction (Fig. 8b). Triggered by electrochemical stimuli, the SEM images show that the membranes have a visibly higher density of openings in the oxidized form (Fig. 8c) and a higher density of closed cells in the reduced state (Fig. 8d). Chemical oxidation/reduction also induces a similar, but much faster structure change. The obtained redox porous membrane exhibited reversible permeability induced by oxidization and reduction of PFS, and thus changes in the interconnectivity between pores. The average flow rate of pure water under 5 kPa pressure was demonstrated to be 0.092 ± 0.004 mL cm^–2^ s^–1^ in the oxidized state and 0.064 ± 0.005 mL cm^–2^ s^–1^ in the reduced state. Such membranes with breathing pores on command may show great application potential in gated filtration, catalysis, and controlled release.

## Room temperature porous ILs

**Fig. 9:**
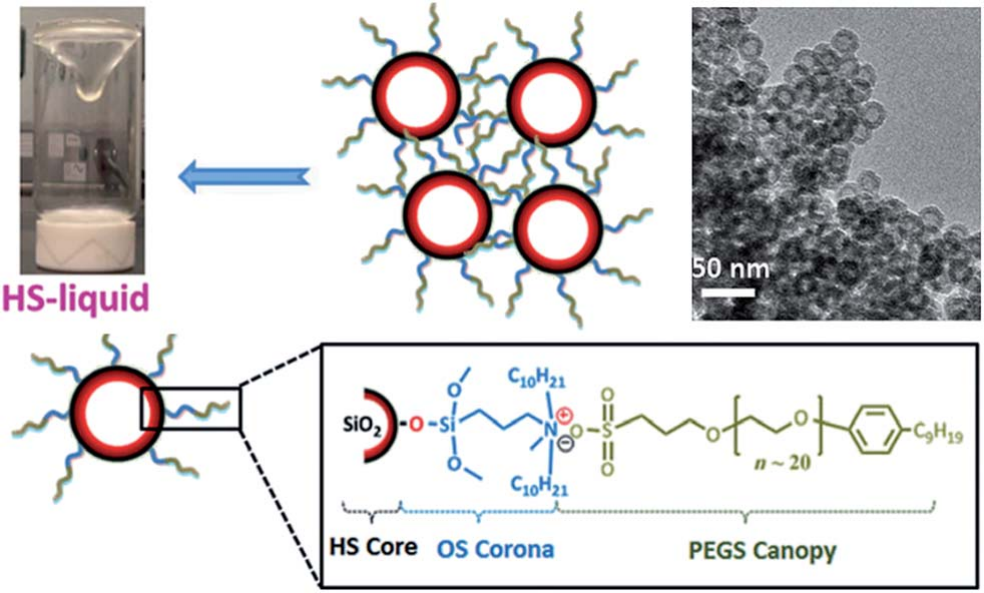
Room temperature porous IL obtained by surface engineering of hollow silica spheres with ionic corona and canopy species. Reproduced with permission from ref. 47 Copyright 2015, Wiley.

The general conclusion drawn from the above discussion is that porosity is a characteristic that is normally associated only with the solid ionic polymers. To get a “real”porous IL is undoubtedly difficult because it is initially counter-intuitive that well-defined pores, as seen in solids, could exist in the liquid state. However, very recently, Dai *et al.* made the impossible possible by surface engineering of hollow silica spheres with suitable ionic corona and canopy species.^47^ The key feature for this synthesis is the use of hollow silica (HS) spheres with microporous shells as the core particles; their shells essentially function as molecular sieves to block species larger than 1.9 nm and prevent the fluid medium from self-filling the cavities. A positively charged organosilane (OS) moiety with molecular size approximately 2.0 nm was used as the corona for surface modification. Then the chloride counteranion was replaced by a negative poly(ethylene glycol)-tailed sulfonate (PEGS) canopy, yielding an optically transparent IL (denoted as HS-liquid, Fig. 9) at room temperature. The intrinsic porosity was confirmed by both transmission electron microscopy (TEM) and N_2_-sorption isotherms. It should be noted that this room temperature IL is a type I porous liquid according to the classification proposed by James *et al.* based on the nature of the host systems,^48^ and is quite distinct from that of conventional colloidal suspensions, where the particles and solvents are physically distinct entities. Interestingly, the resultant porous IL can work as a promising candidate for gas separation, for which the liquid-like polymeric matrices and the empty cavities can function as a separation medium and gas transport pathway, respectively. The experiment of CO_2_ permeability indicates that the HS-liquid can accelerate gas transport in the dense liquid state as compared to pure PEGS, while keeping the CO_2_/N_2_ selectivity unchanged. This concept can be extended to the fabrication of other advanced porous liquids, and the properties of this hybrid system can be further optimized by virtue of the good tunability of the ionic motifs.

## Conclusions

In summary, solidification of fluidic ILs into porous materials has succeeded through either template- (hard or soft) or template-free methods. A series of ordered or disordered porous ILs were ultimately obtained with micro-, meso-, or macro-pores, or a hierarchical architecture. Typically, *in situ* polymerization of monomeric ILs in the presence of a template yields porous ILs, although tedious and complicated synthetic procedures are unavoidable. In contrast, template-free methods, especially the facile synthesis of porous ILs *via* simple ionic complexation in a very short time, are easy to implement and the production can be readily scaled up without templates or additional steps involving polymerization and template removal. Undoubtedly, the ionic complexation-based approach is specific to ILs consisting of discrete cations and anions. Depending on the synthetic procedures, either porous powders or membranes can be readily constructed. Considering the ionic features offered by porous ILs, a variety of novel functional materials could be built up by finely adjusting the IL structure, framework composition, porous architecture, *etc.* Porous ILs can be directly employed as a versatile platform to fabricate materials that are more advanced by decorating them with other functional moieties such as catalytically active anions or redox cations. These distinct features allow for some immediate applications such as catalysis, CO_2_ adsorption, separations, sensors, and actuators. Particularly, the porous IL membranes may open new frontiers for membrane sciences. For example, the templated ordered 3D macroporous IL photonic membranes can be directly used for optical sensing, while the disordered macroporous IL membranes obtained from ionic complexation exhibited great potential as actuators in response to organic vapors. Different from the solid porous ILs, it is believed that the concept of room temperature porous ILs obtained by a facile synthetic strategy could open up new opportunities for preparation of ionic and porous liquids with attractive properties that have never been achieved for porous solid ionic materials and porous liquid molecular materials.

The research on porous ILs is full of potential for the fields of IL chemistry, polymer sciences, and porous materials, but is still in its infancy. First, most of the obtained ionic skeletons consist of mainly large random mesopores and macropores with small surface areas. The development of strategies to produce highly porous ILs with controlled pore structures involving ordered or narrowly distributed mesopores remains a required breakthrough. In addition, considering the high ionic conductivities, wide potential windows, and excellent porosity, application of porous ionic networks as a new subclass of promising solid polyelectrolytes for electrochemical applications (*e.g.*, batteries, fuel cells, and capacitors) is also an issue that deserves further investigation by electrochemists. Another limiting point is that in most cases the variation and tunability of porous ILs is realized by anion exchange, while the construction of porous ILs with anionic polymer backbones has been explored less.
